# Algorithms: simultaneous error-correction and rooting for gene tree reconciliation and the gene duplication problem

**DOI:** 10.1186/1471-2105-13-S10-S14

**Published:** 2012-06-25

**Authors:** Pawel Górecki, Oliver Eulenstein

**Affiliations:** 1 Institute of Informatics, University of Warsaw, Warsaw, 02-097, Poland; 2 Department of Computer Science, Iowa State University, Ames, 50011, USA

## Abstract

**Background:**

Evolutionary methods are increasingly challenged by the wealth of fast growing resources of genomic sequence information. Evolutionary events, like gene duplication, loss, and deep coalescence, account more then ever for incongruence between gene trees and the actual species tree. Gene tree reconciliation is addressing this fundamental problem by invoking the minimum number of gene duplication and losses that reconcile a rooted gene tree with a rooted species tree. However, the reconciliation process is highly sensitive to topological error or wrong rooting of the gene tree, a condition that is not met by most gene trees in practice. Thus, despite the promises of gene tree reconciliation, its applicability in practice is severely limited.

**Results:**

We introduce the problem of reconciling unrooted and erroneous gene trees by simultaneously rooting and error-correcting them, and describe an efficient algorithm for this problem. Moreover, we introduce an error-corrected version of the gene duplication problem, a standard application of gene tree reconciliation. We introduce an effective heuristic for our error-corrected version of the gene duplication problem, given that the original version of this problem is NP-hard. Our experimental results suggest that our error-correcting approaches for unrooted input trees can significantly improve on the accuracy of gene tree reconciliation, and the species tree inference under the gene duplication problem. Furthermore, the efficiency of our algorithm for error-correcting reconciliation is capable of handling truly large-scale phylogenetic studies.

**Conclusions:**

Our presented error-correction approach is a crucial step towards making gene tree reconciliation more robust, and thus to improve on the accuracy of applications that fundamentally rely on gene tree reconciliation, like the inference of gene-duplication supertrees.

## Background

**Figure 1:**
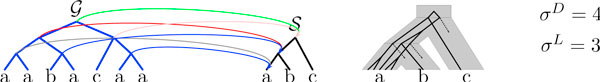
**Rooted reconciliation**. An lca-mapping *M *from the gene tree  into the species tree  and the corresponding embedding. *M *is shown for the internal nodes of .

The wealth of newly sequenced genomes has provided us with an unprecedented resource of information for phylogenetic studies that will have extensive implications for a host of issues in biology, ecology, and medicine, and promise even more. Yet, before such phylogenies can be reliably inferred, challenging problems that came along with the newly sequenced genomes have to be overcome. Evolutionary biologists have long realized that gene-duplication and subsequent loss, a fundamental evolutionary process [1], can largely obfuscate phylogenetic inference [2]. Gene-duplication can form complex evolutionary histories of genes, called gene trees, whose topologies are traditionally used to derive species trees. This approach relies on the assumption that the topologies from gene trees are consistent with the topology of the species tree. However, frequently genes that evolve from different copies of ancestral gene-duplications can become extinct and result in gene trees with correct topologies that are inconsistent with the topology of the actual species tree (see Figure 1). In many such cases phylogenetic information from the gene trees is indispensable and may still be recovered using gene tree reconciliation.

### Related work

Gene tree reconciliation is a well-studied method for resolving topological inconsistencies between a gene tree and a trusted species tree [2-7]. Inconsistencies are resolved by invoking gene-duplication and loss events that reconcile the gene tree to be consistent with the actual species tree. Such events do not only reconcile gene trees, but also lay foundation for a variety of evolutionary applications including ortholog/paralog annotation of genes, locating episodes of gene-duplications in species trees [8-10], reconstructing domain decompositions [11], and species supertree construction [8,12-14].

A major problem in the application of gene tree reconciliation is its high sensitivity to error-prone gene trees. Even seemingly insignificant errors can largely mislead the reconciliation process and, typically undetected, infer incorrect phylogenies (e.g., [7,15]). Errors in gene trees are often topological errors and rooting errors. Topological error results in an incorrect topology of the gene tree that can be caused by the inference process (e.g. noise in the underlying sequence data) or the inference method itself (e.g. heuristic results). This problem has been addressed for rooted gene trees by 'correcting the error'; that is, editing the given tree such that the number of invoked gene-duplications and losses is minimized [16,17]. However, most inference methods used in practice return only unrooted gene trees (e.g. parsimony and maximum likelihood based methods) that have to be rooted for the gene tree reconciliation process. Rooting error is a wrongly chosen root in an unrooted gene tree. Whereas rooting can be typically achieved in species trees by outgroup analysis, this approach may not be possible for gene trees if there is a history of gene duplication and loss [7]. Other rooting approaches like midpoint rooting or molecular clock rooting assume a constant rate of evolution that is often unrealistic. However, rooting problems can be bypassed by identifying roots that minimize the invoked number of gene duplications and losses [7,16-19].

In summary, even small topological error or a slightly misplaced root can incorrectly identify enormous numbers of gene duplications and losses, and therefore largely mislead the reconciliation process. Therefore, gene tree reconciliation requires gene trees that are free of error and correctly rooted at the same time [5]. However, as previous work has incorporated topological error-correction only separately from correctly rooting gene trees into the reconciliation process [16,18], this process can still be misled.

### Our contribution

We address the problem of reconciling erroneous and unrooted gene trees by error-correcting and rooting them at the same time. Solving this problem efficiently is a crucial step towards making gene tree reconciliation more robust, and thus to improve on the accuracy of applications that rely on gene tree reconciliation like the construction of gene-duplication supertrees. We introduce the problem and design an efficient algorithm that facilitates a much more precise gene tree reconciliation, even for large-scale data sets. Our algorithm detects and corrects errors in unrooted gene trees, and thus we avoid the biologists' difficulty and uncertainty of handling erroneous gene trees and correctly rooting them. The presented experimental results suggest that our novel reconciliation algorithms can identify and correct topological error in unrooted input gene trees, and at the same time root them optimally.

Our algorithm is designed to search for the correct and rooted tree of a given unrooted tree in local search neighborhoods of the given tree. The size of these neighborhoods is described by a positive integer *k *that allows to fine-tune the search. While in theory *k *can be large it is assumed that gene trees have only small topological error, which typically can be captured by small values of *k*. For a fixed but freely choosable integer *k *the runtime of our algorithm is *O*(*l^k ^*+ max(*n, m*)), where *n *and *m *is the size of the gene tree and species tree respectively, and *l *is the number of edges in the gene tree that potentially contain an error (such edges will be called *weak*). Thus, for a small error, which is expressed by *k *= 1, our algorithm runs in linear time. Our experiments show that error-correction runs of the algorithm for *k *= 3 are still possible even for trees with large number of weak edges (e.g., *l *= 200) on a standard workstation configuration.

Further, we address the problem of constructing rooted supertrees by reconciling unrooted and erroneous gene trees with assigned weak edges, a key problem in illuminating the role and effect of gene duplication and loss in shaping the evolution of organisms. We introduce the problem and develop an effective local search heuristic that makes the construction of more accurate supertrees possible and allows a much better postulation of gene duplication histories. Our experimental results demonstrate that our approach is effective in identifying gene duplication histories given erroneous gene trees and producing more accurate supertrees under gene tree reconciliation.

### Duplication-loss model

We introduce the fundamentals of the classical duplication-loss model. Our definitions are mostly adopted from [18]. For a more detailed introduction to the duplication-loss model we refer the interested reader to [2,5,10,20].

Let ℐ be the set of species consisting of *N *> 0 elements. The *unrooted gene tree *is an undirected acyclic graph in which each node has degree 3 (internal nodes) or 1 (leaves), and the leaves are labeled by the elements from ℐ. A *species tree * is a rooted binary tree with *N *leaves uniquely labeled by the elements from ℐ. In some cases, a node of a tree will be referred by "cluster" of labels of its subtree leaves. For instance, a species tree (*a*, (*b, c*)) has 5 nodes denoted by: *a, b, c, bc *and *abc*. *A rooted gene tree *is a rooted binary tree with leaves labeled by the elements from ℐ. The internal nodes of a tree *T *we denote by int(*T*).

Let $S=\left\langle V_{S},E_{S}\right\rangle$ be a *species tree*.  can be viewed as an upper semilattice with + a binary least upper bound operation and ⊤ the top element, that is, the root. In particular for *a*, $b\in V_{S}$, *a *<*b *means that *a *and *b *are on the same path from the root, with *b *being closer to the root than *a*. We define the *comparability predicate D*(*a, b*) = 1, if *a *≤ *b *or *b *≤ *a *and *D*(*a, b*) = 0, when *a *and *b *are incomparable. The *distance function ρ*(*a, b*) is used to denote the number of edges on the unique (non-directed) path connecting *a *and *b*.

We call distinct nodes *a*, $b\in V_{S}$*siblings *when *a *+ *b *is a parent of *a *and *b*. For *a*, $b\in V_{S}$ let **Sb**(*a, b*) be the set of nodes defined by the following recurrent rule: **(i) Sb**(*a, b*) = ∅ if *a *= *b *or *a *and *b *are siblings, **(ii) Sb**(*a, b*) = {*c*} ∪ **Sb**(*a *+ *c, b*), if *a *<*b *or *a *+ *c *<*a *+ *b*; here *c *is the sibling of *a*, and **(iii) Sb**(*a, b*) = **Sb**(*b, a*) otherwise.

By *L*(*a, b*) we denote the number of elements in **Sb**(*a, b*). Observe that *L*(*a, b*) = *ρ*(*a, b*) - 2 · (1 - *D*(*a, b*)). Let $M:V_{G}\to V_{S}$ be the *least common ancestor (lca) mapping*, from rooted  into  that preserves the labeling of the leaves. Formally, if *v *is a leaf in  then *M*(*v*) is the node in  labeled by the label of *v*. If *v *is internal node in  with two children *a, b*, then *M*(*v*) = *M*(*a*) + *M*(*b*). An example is depicted in Figure 1.

In this general setting let us assume that we are given a *cost function *$\xi :V_{G}\times V_{S}\to R$ which for all nodes $v\in V_{G}$, $a\in V_{S}$ assigns a real *ξ*(*v, a*) representing a contribution to node *a *which comes from *v *when reconciling  with . Having *ξ *we can define $k\left(v\right)=\sum_{a}\xi \left(v,\;a\right)$ to be a total contribution from *v *in the reconciliation of  with . We call *κ *a *contribution *function. Finally, $\sigma =\sum_{v}k\left(v\right)$ is the total cost of reconciliation of  with .

Now we present examples of cost functions that are used in the duplication model. We assume that if *v *is an internal node in  then *w*_1 _and *w*_2 _are its children. The *Duplication cost *function is defined as follows: *ξ^D^*(*v, a*) = 1 if v∈int(G) and *M*(*v*) = *M*(*w_i_*) = *a *for some *i*, and *ξ^D^*(*v, a*) = 0 otherwise. The *Loss cost *function: *ξ^L^*(*v, a*) = 1 if v∈int(G) and *a *∈ **Sb **(*M*(*w*_1_), *M*(*w*_2_)), and *ξ^L^*(*v, a*) = 0 otherwise. It can be proved that if v∈int(G) then *κ^D^*(*v*) = *D*(*M*(*w*_1_), *M*(*w*_2_)) and *κ^L ^*(*v*) = *L*(*M*(*w*_1_), *M*(*w*_2_)) (in both cases 0 if *v *is a leaf).

The *Duplication cost *function is defined as follows: *ξ^D^*(*v, a*) = 1 if v∈int(G) and *M*(*v*) = *M*(*w_i_*) = *a *for some *i*, and *ξ^D^*(*v, a*) = 0 otherwise. Loss cost function: *ξ^L^*(*v, a*) = 1 if v∈int(G) and *a *∈ **Sb**(*M*(*w*_1_), *M*(*w*_2_)), and *ξ^L^*(*v, a*) = 0 otherwise. It can be proved that if v∈int(G) then *κ^D^*(*v*) = *D*(*M*(*w*_1_), *M*(*w*_2_)) and *κ^L^*(*v*) = *L*(*M*(*w*_1_), *M*(*w*_2_)) (in both cases 0 if *v *is a leaf).

Observe that a node $v\in V_{G}$ is called a duplication [4,13] if *κ^D^*(*v*) = 1. Moreover, *κ^L^*(*v*) = *l*(*v*), where *l*(*v*) is the number of gene losses associated to *v*. It can be proved that *σ^D ^*and *σ^L ^*are the minimal number of gene duplications and gene losses (respectively) required to reconcile (or to embed)  with . Please refer to [18] for more details. The example of an embedding is depicted in Figure 1.

### Introduction to unrooted reconciliation

Here we highlight some results from [18] that are used for the design of our algorithm. From now on, we assume that $G=\langle V_{G},E_{G}{}^{\;}\rangle$ is an unrooted gene tree. We define a rooting of  by selecting an edge $e\in E_{G}$ on which the root is to be placed. Such a rooted tree will be denoted by $G_{e}$, where v_* _is a new node defining the root. To distinguish between rootings of , the symbols defined in previous section for rooted gene trees will be extended by inserting index *e*. Please observe, that the mapping of the root of $G_{e}$ is independent of *e*. Without loss of generality the following is assumed: **(A1) ** and  have at least one internal node and **(A2) ***M_e_*(v_*_)=⊤; that is, the root of every rooting is mapped into the root of  (we may always consider the subtree of the species tree rooted in *M_e_*(v_*_) with no change of the cost).

First, we transform  into a directed graph $\hat{G}=\left\langle V_{G},\hat{E_{G}}\right\rangle$ where $\hat{E_{G}}=\left\{\left\langle v,\;w\right\rangle |\;\left\{v,\;w\right\}\in E_{G}\right\}$. In other words each edge 〈*v, w*〉 in  is replaced in $\hat{G}$ by a pair of directed edges 〈*v, w*〉 and 〈*w, v*〉.

Edges in $\hat{G}$ are labeled by nodes of  as follows. If $v\in V_{G}$ is a leaf labeled by *a*, then the edge $\left\langle v,w\right\rangle \in \hat{E_{G}}$ is labeled by *a*. When *v *is an internal node in $\hat{G}$ we assume that 〈*w*_1_, *v*〉 and 〈*w*_2_, *v*〉 are labeled by *b*_1 _and *b*_2_, respectively. Then the edge $\left\langle v,w_{3}\right\rangle \in \hat{E_{G}},$ such that *w*_3 _≠ *w*_1 _and *w*_3 _≠ *w*_2 _is labeled by *b*_1 _+ *b*_2_. Such labeling will be used to explore mappings of rootings of . An edge {*v, w*} in  is called *asymmetric *if exactly one of the labels of 〈*v, w*〉 and 〈*w, v*〉 in $\hat{G}$ is equal to ⊤, otherwise it is called *symmetric*.

**Figure 2:**
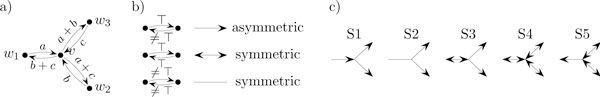
**Unrooted reconciliation**. a) A star in $\hat{G}$. b) Types of edges. c) All possible types of stars. We use simplified notation instead of the full topology.

Every internal node *v*, and its neighbors in $\hat{G}$ define a subtree of $\hat{E_{G}}$, called a *star *with a center *v*, as depicted in Figure 2. The edges 〈*v, w_i_*〉 are called *outgoing*, while the edges 〈*w_i_, v*〉 are called *incoming*. We will refer to the undirected edge {*v, w_i_*} as *e_i_*, for *i *= 1, 2, 3.

The are several types of possible star topologies based on the labeling (for proofs and details see [18]): (S1) a star has one incoming edge labeled by ⊤ and two outgoing edges labeled ⊤ and these edges are connected to the three siblings of the center, (S2) a star has exactly two outgoing edges labeled by ⊤, (S3) a star has all outgoing edges and exactly one incoming edgd labeled by ⊤, (S4) a star has all edges labelled by *top*, and (S5) a star has all outgoing edges and exactly two incoming edges labeled by ⊤. Figure 2 illustrates the star topologies.

In summary stars are basic 'puzzle-like' units that can be used to assemble them into unrooted gene trees. However, not all star compositions represent a gene tree. For instance, there is no gene tree with 3 stars of type S2. It follows from [18] (see Lemma 4) that we need the following additional condition: (C1) if a gene tree has two stars of type S2 then they share a common edge.

Now we overview the main result of [18] (see Theorem 1 for more details). Let  be a species tree and  be unrooted gene tree. The set of optimal edges, that is, candidates for best rootings, is defined as follows: $Min_{G}=\left\{e\in E_{G}|\sigma_{e}^{M_{\alpha ,\beta }}\text{ is minimal}\right\}$, where $\sigma_{e}^{M_{\alpha ,\beta }}$is the total cost for the weighted mutation cost defined by $\xi_{e}^{M_{\alpha ,\beta }}\left(v,\;a\right)=\alpha \;\cdot \;\xi_{e}^{D}\left(v,\;a\right)+\beta \;\cdot \;\xi_{e}^{L}\left(v,\;a\right)$, *e *is an edge in  and *α, β *are two positive reals. Then **(M1) **if $|Min_{G}|>1$, then $Min_{G}$ consists of all edges present in all stars of type S4 or S5, **(M2) **if $|Min_{G}|=1$, then $Min_{G}$ contains exactly one symmetric edge that is present in star of type S2 or S3. From the above statements, (C1) and star topologies we can easily determine $Min_{G}$. More precisely, the star edges outside $Min_{G}$ are asymmetric and share the same direction. Thus, to find an optimal edge it is sufficient to follow the direction of non ⊤ edges in $\hat{G}$.

Now we summarize the time complexity of this procedure. It follows from [21] that a single lca-query (that, is *a *+ *b *for nodes *a *and *b *in ) can be computed in constant time after an initial preprocessing step requiring O(|S|) time. Other structures like $\hat{G}$ with the labeling can be computed in O(|G|) time. The same complexity has the procedure of finding an optimal edge in . In summary an optimal edge/rooting and the minimal cost can be computed in linear time. See [18] for more details and other properties.

## Methods

**Figure 3:**
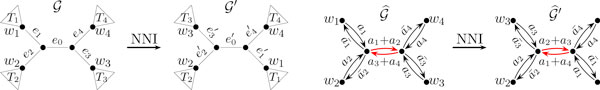
**NNI**. A single NNI on  and $\hat{G}$. On the left *e*_i _and $e_{i}^{\prime }$ (for *i *= 0, ... , 4) denote edges in  and its NNI-neighbor $G^{\prime }$, respectively. On the right each node *a_i _*denote the labeling of edges in $\hat{G}$. Notation ${ā}_{i}$ denote the lca-mapping of complementary subtrees, for instance, ${ā}_{3}=a_{1}+a_{2}+a_{4}$, etc. For brevity, we omit each subtree *T_i _*attached to *w_i _*in the left diagram.

First we describe our algorithm for computing the optimal cost and the set of optimal edges after one nearest neighbor interchange (NNI) operation performed on an unrooted gene tree, and then extend it to a general case with *k *NNI operations. For the definition of NNI please refer to Def. 1 and Figure 3.

### Algorithm

Now we show that a single NNI operation can be completed in constant time if all structures required for computing the optimal rootings are already constructed. First, let us assume that the following is given: (a) two positive reals *α *and *β*, a species tree , (b) lca structure for  that allows to answer lca-queries in constant time, (c) an unrooted gene tree , (d) $\hat{G}$ with the labeling of edges, (e) $Min_{G}$ - the set of optimal edges, and (f) *σ *- the minimal total weighted mutation cost. As observed in the previous section (b),(d)-(f) can be computed in O(max(|S|,|G|)). Now we show that (c)-(f) can be computed in constant time after a single NNI operation.

*NNI operation (c) and the update of lca-mappings (d)*.

**Definition 1**. *(Single NNI operation) An NNI operation transforms a gene tree $G=\left(\left(T_{1},T_{2}\right),\left(T_{3},T_{4}\right)\right)$ into *$G^{\prime }=\left(\left(T_{2},T_{3}\right),\left(T_{1},T_{4}\right)\right)$*, where T_i_-s are (rooted) subtrees of **. The edge that connects the roots of *(*T*_1_, *T*_2_) *and *(*T*_3_, *T*_4_) *in **is denoted by e*_0 _*and called the *center edge. *For each i *= 1, 2, 3, 4 *we assume the following: w_i _is the root of T_i_, e_i _is the edge connecting w_i _with e*_0 _*and a_i _is the lca-mapping of T_i_*. *Similarly, we define the *center edge $e_{0}^{\prime }$*and $e_{i}^{\prime }$ in *$G^{\prime }$.

An NNI operation is depicted in Figure 3 with the transformation of $\hat{G}$ into $\hat{G^{\prime }}$. The notation will be used from now on. Note that there is a second NNI operation, when  is replaced with ((*T*_1_, *T*_3_), (*T*_2_, *T*_4_)). However, it can be easily defined and therefore it is omitted here. Observe that the NNI operation (without updating of lca-mappings) can be performed in constant time for both trees.

The right part of Figure 3 depicts the transformation of $\hat{G}$. Observe that the labels of the incoming and outgoing edges attached to each *w_i _*in $\hat{G}$ do not change during this operation. Lemma 1 follows directly from this observation.

**Lemma 1**. *An NNI operation changes only the labels of the center edge*.

We conclude that updating $\hat{G}$ requires only two lca-queries, and therefore can be performed in constant time.

*Reconstruction of optimal edges (e)*. We analyze the changes of the optimal set of edges $Min_{G}$. To this end we consider a number of cases depending on the relation between the optimal set of edges and the set of edges, incident to the nodes of the center edge. Let $C_{G}={\left\{e_{i}\right\}}_{i=0,\ldots ,4}$.

For convenience, assume that the NNI operation replaces *e_i _*with $e_{i}^{\prime }$ as indicated in Figure 3. We call two disjoint edges from $C_{G}$*semi-alternating *if they share a common node after the NNI operation. In Figure 3 {*e*_1_, *e*_4_} and {*e*_2_, *e*_3_} are semi-alternating. For two edges *a *and *b *that are incident to the same node let ⋆(*a, b*) be the set of three edges defining the unique star that contains *a *and *b*.

**Lemma 2**. *Assuming that e_i _is replaced by $e_{i}^{\prime }$ after the NNI operation the set of optimal edges does not require additional changes if and only if one of the following conditions is satisfied:*

$\left(EQ1\right)\;Min_{G}\cap C_{G}=\emptyset ,$

$\left(EQ2\right)\;Min_{G}⊇C_{G}$*and each pair of semi-alternating edges contains at least one symmetric edge*,

**$\left(EQ3\right)\;Min_{G}$***consists of only the center edge*,

$\left(EQ4\right)\;Min_{G}\cap C_{G}=\left\{e_{i}\right\}$*for some i >*0 *and the center is asymmetric after the NNI operation*.

Proof: (EQ1) All edges in $C_{G}$ are asymmetric (2 stars S1). Then, after the NNI operation $e_{0}^{\prime }$ is asymmetric and ($C_{G^{\prime }}$ has 2 stars S1). (EQ2) $C_{G}$ consists of 2 stars of type S4/S5 and at most two asymmetric edges. It follows from EQ2 that the asymmetric edges in $C_{G^{\prime }}$ cannot form a star of type other than S5. Together with M1 it follows that $C_{G^{\prime }}$ is optimal. (EQ3) By M1 the center is symmetric in . It remains symmetric after NNI. From C1 and M2, $Min_{G^{\prime }}$ consists of the center edge. (EQ4) Note, that the type of $⋆\left(e_{i}^{\prime },e_{0}^{\prime }\right)$ is *S*1, *S*2 or *S*3.

**Lemma 3 **(NE1). *If *$Min_{G}⊇C_{G}$*and there exists a pair *{*e_i_ , e_j_*} *of asymmetric semi-alternating edges, then *$Mi{n^{\prime }}_{G}=Min_{G}\backslash C_{G}\cup \left(C_{G^{\prime }}\backslash \left\{e_{i}^{\prime },\;e_{j}^{\prime }\right\}\right)$.

Proof: The type of $⋆\left(e_{i}^{\prime },e_{j}^{\prime }\right)$ is S1 or S3 and the other star has type S4 or S5. By M2 $e_{i}^{\prime }$ and $e_{j}^{\prime }$ are not optimal.

**Lemma 4 **(NE2). *If $Min_{G}\cap C_{G}=\left\{e_{i}\right\}$ for some i >*0 *and the center is symmetric after the NNI operation then *$Mi{n^{\prime }}_{G}=Min_{G}\backslash \left\{e_{i}\right\}\cup ⋆\left(e_{0}^{\prime },\;e_{j}^{\prime }\right)$.

Proof: In this case $e_{0}^{\prime }$ has two arrows and $⋆\left(e_{0}^{\prime },e_{i}^{\prime }\right)$ is of type S5.

**Lemma 5**. *Assume that $Min_{G}\cap C_{G}=\left\{e_{0},e_{i},e_{j}\right\}$ , where i ≠ *0,

**(NE3) ***If both e_i _and e_j _are symmetric then *$Min_{G^{\prime }}=Min_{G}\backslash C_{G}\cup C_{G^{\prime }}$,

**(NE4) ***If e_j _is asymmetric and $e_{0}^{\prime }$ is symmetric then *$Min_{G^{\prime }}=Min_{G}\backslash C_{G}\cup ⋆\left(e_{0}^{\prime },\;e_{i}^{\prime }\right)$.

**(NE5) ***If both e_j _and $e_{0}^{\prime }$ are asymmetric then $Min_{G^{\prime }}=Min_{G}\backslash C_{G}\cup \left\{e_{i}^{\prime }\right\}$*.

Proof: Note that {*e*_0_, *e_i_ , e_j_*} must be a star in $G\cdot \left(\text{NE}3\right)⋆\left(e_{i},e_{j}\right)$ has type S4 or S5. After the transformation the two stars $⋆\left(e_{0}^{\prime },e_{i}^{\prime }\right)$ and $⋆\left(e_{0}^{\prime },e_{j}^{\prime }\right)$ have type S5. Both are optimal in $G^{\prime }\cdot \left(\text{NE4}\right)⋆\left(e_{i},e_{j}\right)$ has type S5. After the transformation $⋆\left(e_{0}^{\prime },e_{i}^{\prime }\right)$ has type S5 and $⋆\left(e_{0}^{\prime },e_{j}^{\prime }\right)$ has type S3. Only the first is optimal in $G^{\prime }\cdot \left(\text{NE5}\right)⋆\left(e_{i},e_{j}\right)$ has type S5 while the other star in $C_{G}$ has type S3. After the transformation only $e_{i}^{\prime }$ remains symmetric in $C_{G^{\prime }}$ therefore it is the only optimal edge in $C_{G^{\prime }}$.

*Computing the optimal cost (f)*. Observe that from Lemmas 2-5 at least one optimal edge remains optimal after the NNI operation. Therefore, to compute the difference in costs between optimal rootings of  and $G^{\prime }$ we start with the cost analysis for the rootings of such edge.

First, we introduce a function for computing the cost differences. Consider three nodes *x, y, z *of some rooted gene tree such that *x *and *y *are siblings and the parent of them (denoted by *xy*), is a sibling of *z*. In other words we can denote this subtree by ((*x, y*), *z*). Then, the partial contribution of ((*x, y*), *z*) to the total weighted mutation cost can be described as follows: $\sum_{a\in S}\alpha *\left(\xi^{D}\left(xy,\;a\right)+\xi^{D}\left(xyz,\;a\right)\right)+\beta *\left(\xi^{L}\left(xy,\;a\right)+\xi^{L}\left(xyz,\;a\right)\right)$. Assume that *x, y *and *z *are mapped into *a, b *and *c *(from the species tree), respectively. It can be proved from the defnition of *ξ^D ^*and *ξ^L ^*that the above contribution equals: *ϕ*(*a, b, c*) = α * (*D*(*a, b*) + *D*(*a *+ *b, c*)) + β * (*L*(*a, b*) + *L*(*a *+ *b, c*)). Now, assume that a single NNI operation changes ((*x, y*), *z*)) into (*x*, (*y, z*)). It should be clear that the cost difference is given by: Δ_3_(*a, b, c*) = *ϕ*(*c, b, a*) - *ϕ*(*a, b, c*). Similarly, we can define a cost difference when a single NNI operation changes ((*x, y*), (*z, v*)) into ((*x, v*), (*y, z*)). Assume, that *v *is mapped into *d*. Then, the cost contribution of the first subtree is *ϕ*'(*a, b, c, d*) = *ϕ*(*a, b, c *+ *d*) + α * (*D*(*c, d*) + β * *L*(*c, d*). The cost difference is given by: Δ_4_(*a, b, c, d*) = *ϕ*'(*a, d, b, c*) - *ϕ*'(*a, b, c, d*).

**Lemma 6**. *If the center edge is optimal and remains optimal after the NNI operation then the cost difference equals *Δ_4_(*a*_1_, *a*_2_, *a*_3_, *a*_4_)*, where a_i _(for i *= 1, 2, 3, 4*) is the mapping as indicated in *Figure 3.

As mentioned the above lemma can be proved by comparing the rootings placed on the center edges in  and $G^{\prime }$. Lemma 6 gives a solution for cases: EQ2, EQ3, NE1 and NE3. The next lemma gives a solution for the remaining cases.

**Lemma 7**. *If for some i >*0 *there exists an optimal edge in T_i _*∪ {*e_i_*} *that remains optimal after the NNI operation (under assumption that e_i _is replaced by $e_{i}^{\prime }$) then the cost difference is *Δ_3_(*a*_4_, *a*_3_, *a*_2_) *if i *= 1, Δ_3_(*a*_3_, *a*_4, _*a*_1_) *if i *= 2, Δ_3_(*a*_2_, *a*_1_, *a*_4_) *if i *= 3 *and *Δ_3_(*a*_1_, *a*_2_, *a*_3_) *if i *= 4.

Similarly to Lemma 6 we can prove Lemma 7 by comparing the rootings of *e_i _*and $e_{i}^{\prime }$.

**Figure 4:**
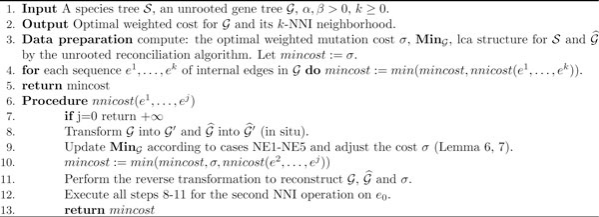
**Algorithm**. Optimal weighted cost for  and its *k*-NNI neighborhood.

*Error correction algorithm*. Finally, we can present the algorithm for computing the optimal weighted mutation cost for a given gene tree and its *k*-NNI neighborhood. See Figure 4 for details. It should be clear that the complexity of this algorithm is $O\left(|G{|}^{k}+\text{max}\left(\left|G|,|S\right|\right)\right)$. We write that a gene tree has *errors *if the optimal cost is computed for one of its NNI variants. Otherwise, we write that a gene tree *does not require corrections*. Please note that it for a special case of *k *= 1, this algorithm is linear in time (see also our preliminary article [22]).

### General reconstruction problems

We present several approaches to problems of error correction and phylogeny reconstruction. Let us assume that $\sigma_{\alpha ,\beta ,k}\left(S,\;G\right)$ is the cost computed by algorithm from Figure 4, where *α, β *> 0, *k *≥ 0,  is a rooted species tree and  is an unrooted gene tree.

**Problem 1 **(*k*NNIC). *Given a rooted species tree **and a set of unrooted gene trees, G compute the total cost *$\sum_{G\in G}\sigma_{\alpha ,\beta ,k}\left(S,\;G\right)$.

The *k*NNIC problem can be solved in polynomial time by an iterative application of our algorithm. Additionally, we can reconstruct the optimal rootings as well as the correct topology of each gene tree. Please note that for *k *= 0 (no error correction), we have the cost inference problem for the reconciliation of an unrooted gene tree with a rooted species tree [18].

**Problem 2 **(*k*NNIST). *Given a set of unrooted gene trees G find the species tree  that minimizes the total cost *$\sum_{G\in G}\sigma_{\alpha ,\beta ,k}\left(S,\;G\right)$.

The complexity of the *k*NNIST problem is unknown. However, similar problems for the duplication model are NP-hard [13]. Therefore we developed heuristics for the *k*NNIST problem to use them in our experiments.

In applications there is typically no need to search over all NNI variants of a gene tree. For instance, a good candidate for an NNI operation is *a weak edge*. A weak edge is usually defined on the basis of its length, where short length indicates weakness. To formalize this property, let us assume that each edge in a gene tree  has length. We call an edge *e *in *weak *if the length of *e *is smaller than *ω*, where *ω *is a non-negative real. Now we can define variants of *k*NNIC and *k*NNIST denoted by *ω*-*k*NNIC and *ω*-*k*NNIST, respectively, where the NNI operations are performed on weak edges only. These straighforward definitions are omitted. Please note that the time complexity of the algorithm with NNIs limited to weak edges is $O\left(l^{k}+\text{max}\left(\left|G|,|S\right|\right)\right)$, where *l *is the number of weak edges in .

### Software

The unrooted reconciliation algorithm [18] and its data structures are implemented in program URec [23]. Our algorithm partially depends on theses data structures and therefore was implemented as a significantly extended version of URec. Additionally, we implemented a hill climbing heuristic to solve *k*NNIST and *ω*-kNNIST.

Software and datasets from our experiments are made freely available through http://bioputer.mimuw.edu.pl/~gorecki/ec.

### Experimental results and discussion

#### Data preparation

First, we inferred 4133 unrooted gene trees with branch lengths from nine yeast genomes contained in the Genolevures 3 data set [24], which contains protein sequences from the following nine yeast species: *C. glabrata *(4957 protein sequences, abbreviation CAGL), *S. cerevisiae *(5396, SACE), *Z. rouxii *(4840, ZYRO), *S. kluyveri *(5074, SAKL), *K. thermotolerans *(4933, KLTH), *K. lactis *(4851, KLLA), *Y. lipolytica *(4781, YALI), *D. hansenii *(5006, DEHA) and *E. gossypii *(4527, ERGO).

**Figure 5:**
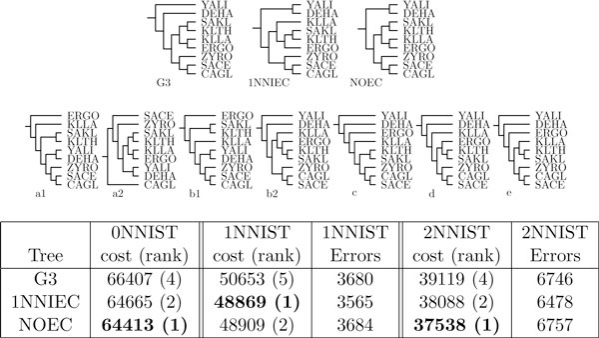
**Yeasts phylogeny**. Species tree topologies. G3 - original phylogeny of Genolevures 3 data set [24]. 1NNIEC - optimal rooted species tree inferred from gene trees with all possible 1-NNI error corrections. NOEC - optimal species tree for the yeast gene trees with no NNI operations (cost 64413, no corrections). Rank denotes a position of a tree on the sorted list of the best trees. The trees below are inferred from other *ω*-*k*NNIST (see next figures). Please note that NOEC, G3, a1 and a2 are rooted variants of the same unrooted tree. Similar property holds for 1NNIEC, b1 and b2.

We aligned the protein sequences of each gene family by using the program TCoffee [25] using the default parameter setting. Then maximum likelihood (unrooted) gene trees were computed from the alignments by using proml from the phylip software package. The original species tree of these yeasts [24], here denoted by G3, is shown in Figure 5.

#### Inferring optimal species trees

The optimal species tree reconstructed with error corrections (1NNIST optimization problem) is depicted in Figure 5 and denoted by 1NNIEC. This tree differs from G3 in the rooting and in the middle clade with KLLA and ERGO. Additionally, we inferred by the heuristic an optimal species tree, denoted here by NOEC, with no error corrections (0NNIST optimization). All the trees from this figure are highly scored in each of the optimization schemas.

#### From weak edges to species trees

In the previous experiment, the NNI operations were performed on almost every gene tree in the optimal solution and with no restrictions on the edges. In order to reconstruct the trees more accurately, we performed experiments for *ω*-*k*NNIST optimization with various *ω *parameters and subsets of gene trees. The filtering of gene trees was determined by an integer *μ *> 0 that defines the maximum number of allowed weak edges in a single gene tree. Each gene tree that did not satisfy such condition was rejected.

**Figure 6:**
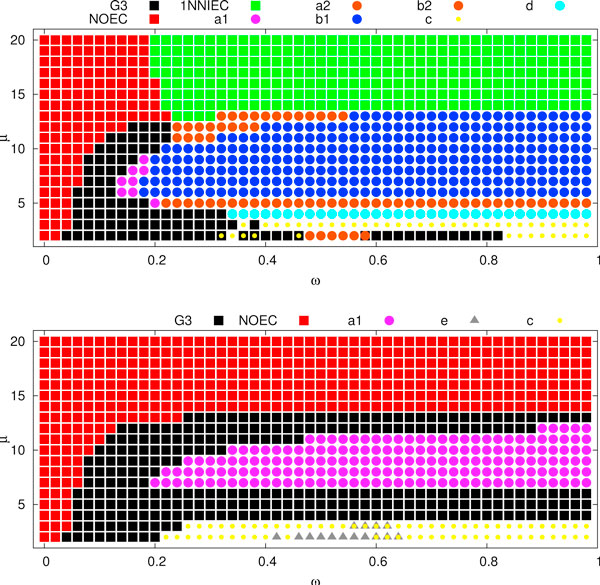
*****ω***-1NNIST and ***ω***-2NNIST experiments**. A summary of *ω*-1NNIST (top) and *ω*-2NNIST experiments (bottom) for *ω *= 0, 0.02, 0.04, ... , 0.98, *μ *= 2, 3, ... , 20. Optimal species trees found by the heuristics. Please note that in some cases two optimal trees were found.

**Figure 7:**
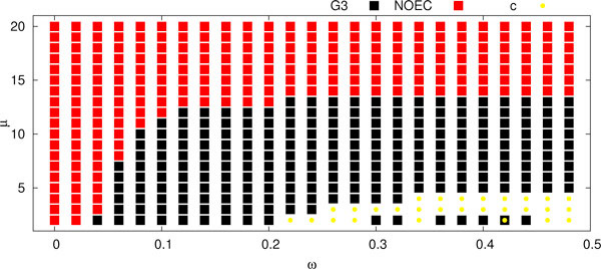
*****ω***-3NNIST experiments**. A summary of *ω*-3NNIST experiments for *ω *= 0, 0.02, 004, ... , 0.48 and *μ *= 2, 3, ... , 20.

**Figure 8:**
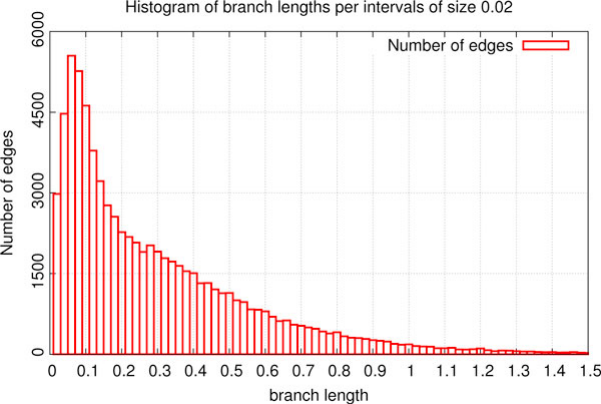
**Branch lengths**. Histogram of branch lengths.

**Figure 9:**
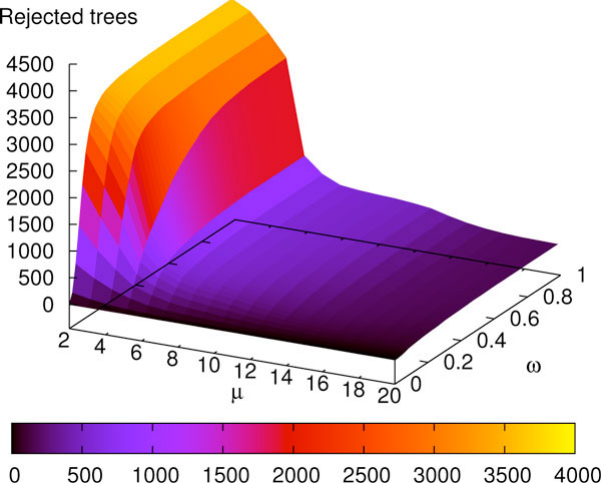
**Rejected gene trees**. The number of rejected trees as a function of *μ *and *ω*.

Figures 6 and 7 depict a summary of error correction experiments for weak edges. For each *ω *and *μ *we performed 20 runs of the *ω*-*k*NNIST heuristic for finding the optimal species tree in the set of gene trees filtered by *μ*. The optimal species trees are depicted in the diagram, where each cell represents the result of a single *ω*-*k*NNIST experiment. We observed that G3, 1NNIEC and NOEC are significantly well represented in the set of optimal species trees in *ω*-1NNIST experiments, while in *ω*-2NNIST and *ω*-3NNIST experiments only G3 and NOEC were detected. Note that the original yeast phylogeny (G3, black squares in Figures 6 and 7) is inferred for *ω *= 0.1-0.2 (in other words approx. 30-40% of edges are weak, see Figure 8) and *μ *≥ 10 in most experiments. In particular for *ω *= 0.15 and *μ *= 10, 364 gene trees were rejected (see Figure 9). These results significantly support the G3 phylogeny. Please note that the results for the standard unrooted reconciliation algorithms without error correction are located in the first column of diagrams (*ω *= 0).

#### From trusted species tree to weak edges in gene trees - automated and manual curation

Assume that the set of unrooted gene trees and the rooted (trusted) species tree  are given. Then we can state the following problem: find *ω *and *μ *such that  is the optimal species tree in *ω*-NNIST problem for the set of gene trees filtered by *μ*. For instance in our dataset, if we assume that G3 is a given correct phylogeny of yeasts, then from the diagrams (Figure 6 and 7) one can determine appropriate values of *ω *and *μ *that yield G3 as optimal. In other words we can automatically determine weak edges by *ω *and filter gene trees by *μ*. This approach can be applied in tree curation procedures to correct errors in an automated way as well as to find candidates (rejected trees) for further manual curation. For instance, in the previous case, when *ω *= 0.1 and *μ *= 10, we have 3164 trees that can be corrected and rooted by our algorithm, while the 364 rejected trees could be candidates for further manual correction.

## Discussion

We present novel theoretical and practical results on the problem of error correction and phylogeny reconstruction. In particular, we describe a polynomial time and space algorithm that simultaneously solves the problem of correction topological errors in unrooted gene trees and the problem of rooting unrooted gene trees. The algorithm allows us to perform efficiently experiments on truly large-scale datasets available for yeast genomes. Our experiments suggest that our algorithm can be used to (i) detect errors, (ii) to infer a correct phylogeny of species under the presence of weak edges in gene trees, and (iii) to help in tree curation procedures.

## Conclusion

We introduced a novel polynomial time algorithm for error-corrected and unrooted gene tree reconciliation. Experiments on yeast genomes suggests that an implementation of our algorithm can greatly improve on the accuracy of gene tree reconciliation, and thus, curate error-prone gene trees. Moreover, we use our error-corrected reconciliation to make the gene duplication problem, a standard application of gene tree reconciliation, more robust. We conjecture that the error-corrected gene duplication problem is intrinsically hard to solve, since the gene duplication problem is already NP-hard. Therefore, we introduced an effective heuristic for error-corrected gene duplication problem. Our experimental results for a wide range of error-correction tests on yeasts phylogeny show that our error-corrected reconciliations result in improved predictions of invoked gene duplication and loss events that then allow to infer more accurate phylogenies.

The presented error correction is based on gene-species tree reconciliation using gene duplication and loss. However, there are other major evolutionary mechanism that infer gene tree topologies that are inconsistent with the actual species tree topology, like horizontal gene transfer and deep coalescence. Gene tree reconciliation using these mechanisms is highly sensitive to topological error, similar to gene tree reconciliation under gene duplication and loss. Future work will focus on the development of algorithms that can also reconcile unrooted and erroneous gene trees using horizontal gene transfer and deep coalescence.

## Competing interests

The authors declare that they have no competing interests.

## Authors' contributions

PG and OE were responsible for algorithm design and writing the paper. PG implemented the programs, and performed the experimental evaluation and the analysis of the results. Both authors read and approved the final manuscript.
